# Natural history of MRI brain volumes in patients with neuronal ceroid lipofuscinosis 3: a sensitive imaging biomarker

**DOI:** 10.1007/s00234-022-03039-z

**Published:** 2022-08-30

**Authors:** Jan-Niklas Hochstein, A. Schulz, M. Nickel, S. Lezius, M. Grosser, J. Fiehler, J. Sedlacik, U. Löbel

**Affiliations:** 1 Department of Diagnostic and Interventional Neuroradiology, University Medical Center Hamburg-Eppendorf, Hamburg, Germany; 2 Department of Pediatrics, University Medical Center Hamburg-Eppendorf, Hamburg, Germany; 3 Department of Biometrics, University Medical Center Hamburg-Eppendorf, Hamburg, Germany; 4 Department of Radiology, Great Ormond Street Hospital for Children, London, UK

We greatly appreciated your interest in our recent publication in Neuroradiology [1] and the comments made by Dr. Ostergaard.

We agree that a more detailed analysis of cortical sub-regions with respect to the clinical presentation would be very interesting and may provide a better understanding of the underlying pathophysiology. This may be particularly true for the optic pathways, where retinal degeneration [2] and secondary cortical atrophy (Wallerian degeneration) may be present earlier compared to other cortical regions. In this respect, diffusion tensor imaging might also be a very useful tool in describing the disease course. However, this may not necessarily yield an improved imaging marker describing disease progression. Splitting up our data will result in a higher variability but not necessarily improve the magnitude of the resulting effect. Small absolute errors in parcellating the cortical regions will have a large effect, because of the small volumes of the investigated sub-regions. The variability would also increase if the data were split into separate age groups since the volume was not always uniformly decreasing for every subject from one scan to the next.

In our publication, we aimed to find and report a robust marker based on MRI alone, which may be used as an endpoint in treatment trials independently from the clinical presentation of the disease. We found that the ROI ‘supratentorial cortical grey matter’ fulfils this requirement. We found a solid and uniform 4.6 ± 0.2% decline in the supratentorial cortical grey matter volume per year. This is a very strong effect, which should be detectable in treatment trials within one year if a well-designed imaging and volumetric analysis protocol with a high test–retest repeatability is employed [3].

Regarding the high variability of grey matter volume with respect to visual scores (0, 1, and 2), it is difficult to definitely conclude that these may be related to different trajectories of cortical atrophy for the occipital cortex and other regions. This would need additional data analysis, and preferably, correlation with a more sensitive novel ophthalmic scale [4]. As this novel score can only be applied prospectively by an NCL expert ophthalmologist, data for the patient cohort studied in this project had been too limited for correlation studies, and this aspect will have to be studied in the future. However, the high variability of grey matter volume in patients with a vision score of 0 is expected as vision loss occurs early in the disease progression and patients are already blind in their early teenage years when other symptoms such as cognitive and motor regression still continue to progress for several years. To date, clinical tools to monitor disease progression in this early stage of the disease had been mostly limited to ophthalmic examination tools—hence the necessity of the development of a novel, refined ophthalmic rating scale [4]. Therefore, our finding of a constant decline of cortical grey matter volume, also in early disease stages, is important as it underlines that brain volumetric analysis can be applied early on to monitor disease progression but also in late stages of the disease when the disease course is more (prevalence of motor and intellectual decline) (5). The observed variability when correlating with the clinical scores is, therefore, not necessarily related to the variability of the MRI data.

We would like to thank Professor Emeritus Alfried Kohlschütter for very helpful discussions in preparing this reply.
